# Supplementary material for: Mortality and major disease risk among migrants of the 1991–2001 Balkan wars to Sweden: A register-based cohort study
Source: PLoS Med. 2020 Dec 1;17(12):e1003392. doi: 10.1371/journal.pmed.1003392 (PMC7707579; doi:10.1371/journal.pmed.1003392)
Supplement: S1 Study Protocol — (DOC) [file pmed.1003392.s001.doc]

**Mortality and major disease risk among Balkan war refugee-immigrants to Sweden in the 1990s: trajectories and identifying factors of resilience**

*Authors (alphabetical order)*: Emily Holmes, Fang Fang, Filip Arnberg, Li Yin, Paul Lichtenstein, Pär Sparén, Unnur Valdimarsdóttir, Weimin Ye

Two non-prespecified analyses are highlighted in red.

**BACKGROUND:**

Due to the multiple exposures to trauma, war-refugees constitute a vulnerable population which may have considerably increased risks of various adverse health outcomes. The traumatic events that these people have experienced and witnessed can include those during the war, on the journey to reach Sweden, via news media (e.g. footage of the war); and violence in their host country. In order to meet the health needs of the enormous number of prospective war-refugees to Europe (e.g. from Syria), we need to better understand the extent of health consequences suffered by previous war-refugee populations.

Sweden currently has the largest intake after Germany of refugees from Syria with estimates of >100,000 refugees in the last year alone (United Nations High Commissioner for Refugees (UNHCR), 2015). As many as half of these refugees arriving in Germany have post-traumatic stress disorder (PTSD) (Tomkiw, 2015), though prevalence rates in Sweden are as yet unknown. Overall, there is therefore an urgent need to better understand the impacts of psychological trauma in the refugee population. ***One way to better understand the mental and physical health consequences for refugees is to examine existing registry data on past refugee populations.***

Poor mental health outcomes for refugees and asylum seekers are well-established. A meta-analysis of factors associated with mental health outcomes in a world-wide sample of refugees indicated overall poorer outcomes for refugees compared to non-refugees (Porter & Haslam, 2005). The world-wide prevalence rate for PTSD in refugees is estimated at 30.6%, and for depression is estimated at 30.8%, with torture and exposure to other traumatic events (e.g. displacement and abuse) being most strongly associated with PTSD (Steel et al., 2009). Studies on Swedish immigrants (refugees and non-refugees) in parallel suggest increased risks of psychiatric disorders and suicide while the increment varies considerably across studies and immigrant groups (Gilliver, Sundquist, Li, & Sundquist, 2014). Yet, recent research from Sweden shows that recently settled refugees are less likely to receive drug treatment for their psychiatric problems compared to Swedish-born residents (Brendler-Lindqvist, Norredam, & Hjern, 2014). In children/young people, exposure to violence has been shown to be a key risk factor for mental health problems, whereas stable settlement and social support in the host country have a positive effect on psychological functioning (Fazel, Reed, Panter-Brick, & Stein). War experiences and their effects on mental health in refugees are associated with increased healthcare costs (e.g. use of healthcare services, medication costs), even many years later (Sabes-Figuera et al., 2012). Re-settlement presents its own challenges; for example, in a Swedish study with refugees from the Middle East, social and economic strain and alienation (as measured by a questionnaire developed to assess resettlement stress) have been linked to symptoms of common mental health disorders and PTSD symptoms (Lindencrona, Ekblad, & Hauff, 2008). ***To what extent functional factors, such as education, income, settlement area and social network modify these risks of mental morbidities remains to be established in a complete follow-up of an entire immigrant-refugee population. Furthermore, understanding trajectories of psychiatric disorders and potential mediating role of these on subsequent risk of major diseases and mortality in refugee populations remains to be elucidated.***

Health consequences for refugees extend beyond mental health to wider physical health outcomes. In a study of refugees and asylum seekers in the Netherlands, 51.7% reported poor general health status, and 47.5% reported more than one chronic health condition (e.g. chronic back pain and severe headaches) (Gerritsen et al., 2006). Further, the physical effects of torture experiences and war violence may be wide-ranging, from bone fractures to head injuries, to contraction of sexually transmitted infections following sexual violence (Burnett & Peel, 2001). With regards to mortality rates, a population-based cohort study in Sweden from 1998 to 2006 found higher rates of mortality due to cardiovascular disease and external causes in refugee men compared to male non-refugee immigrants (Hollander et al., 2012). ***Whether risk of somatic diseases is mediated by psychiatric disorders in refugee populations remains to be firmly established.***

Studies investigating resilience in adult/young refugees have identified a number of social, cognitive and behavioural factors associated with positive outcomes. A review of child and adolescent refugee mental health indicated that resilience to mental health symptoms was associated with factors such as social support, parental wellbeing, emotion-focussed coping strategies (in younger children), and problem-focussed coping strategies and use of distraction (in older children) (Lustig et al., 2004). In Sweden, a study of Iraqi refugee children found that lack of PTSD symptoms was linked to adequate emotional expression, supportive family relations, good peer relations, and prosociality (Daud, af Klinteberg, & Rydelius, 2008). Environment in the host country may be particularly important. A 9-year follow-up study of young refugees in Denmark found that recovery from psychological symptoms (anxiety, sleep problems and sadness) versus persistence of symptoms was determined by the number or types of stressful events that occurred after arrival to the host country (Montgomery, 2010). Further, a 2-year longitudinal survey of refugees in Australia found that permanent protection status (compared to temporary protection status) was associated with reduced mental distress, substantial English language improvement and greater social engagement (Steel et al., 2011). ***Further mapping of structural and functional resilience factors (parental health and social functioning, educational attainment, etc.) among refugee children and young adults is essential for future public health interventions targeted for this vulnerable population.***

The Balkan wars stretched over a whole decade (from 1991-2001); leading to the death of more than 140,000 individuals and displacement of more than 4 million individuals. This war constitutes one of the most severe conflicts in Europe in modern times. Asylum seekers from these areas to Sweden were more than 80,000 in 1992 alone (only recently exceeded by current asylum seekers from Syria) and in 2014 120,000 individuals were living in Sweden from former Yugoslavia. Initial reports indicate high levels of distress and posttraumatic stress symptoms, affecting 30-40% of the population (Roth, Ekblad, & Agren, 2006; Sundquist, Johansson, DeMarinis, Johansson, & Sundquist, 2005) while long term follow-up of other outcomes is needed.

**SPECIFIC AIMS:**

Using registry data the overarching aim of this study is to quantify the risk of mortality by natural and unnatural causes as well as risk of major diseases over time among Balkan refugee-immigrants to Sweden (entering 1991-2002) as compared to native Swedes as well as to other European immigrants entering at the same time. A secondary aim will be to identify structural determinants of favorable vs. non-favorable health trajectories in the refugee population.

*Specifically, we aim to document trajectories of:*

- overall mortality, and cause-specific mortality (using the Causes of Death Register).
- in-patient and outpatient diagnoses for cardiovascular disease, cancer, psychiatric disorders and suicide attempts, and functional somatic impairments, such as fibromyalgia, low back
  pain, tension headache, chronic fatigue, non-cardiac chest pain, irritable
  bowel syndrome (using the nationwide Patient, Prescribed Drug and Cancer Registers).
- stress-related disorders such as post-traumatic stress disorder (PTSD), anxiety and depression, and corresponding psychotropic drug use (using the Patient and Prescribed Drug Registers)
- educational attainment in those of school age years at immigration to Sweden
- employment in the labour market

*Through this project, we will e.g. test the following hypotheses:*

1. That refugee immigrants from the Balkan wars are compared to other European immigrants at increased risks of psychiatric disorders, cardiovascular disease and mortality, and low educational attainment (children) during follow-up.
2. That the risk of psychiatric disorders, particularly stress-related disorders, will be highest in the refugee population right after immigration, relative to non-refugee immigrants, and that this risk increment will decline with time.
3. Predictors of trajectories of resilience (not presenting with psychiatric disorders) vs. morbidity (repeatedly presenting with primary or secondary diagnoses of psychiatric disorders) will be identified in the refugee population as: age at immigration, sex, settlement region, employment, income, health of family/caregivers.
4. That diagnosed psychiatric disorders within a year from immigration increase the risk of subsequent somatic diseases among refugees, particularly cardiovascular disease. Conversely, in terms of resilience we predict that absence of psychiatric disorders (including stress-related disorders) will be associated with better physical health outcomes at later time points, and higher levels of educational attainment and employment.

**METHODS:**

**Aim:** *To quantify the risk of mortality by natural and unnatural causes as well as risk of major diseases among Balkan refugee-immigrants to Sweden (entering 1991-2002) as compared to other European immigrants and native Swedish residents.*

**Design:** We will conduct a register-based, historical cohort study nested in the Swedish registries contrasting mortalities and major disease incidence in refugee-, non-refugee European immigrant populations to Sweden (immigrating 1991-2002) and native Swedish residents.

**Ascertainment of exposure:**

*Exposed population:* Individuals entering Sweden from January 1^st^ 1991 through December 31^st^ 2002 with a country of birth registered as: Albania, Bosnia-Herzegovina, Yugoslavia, Croatia, Macedonia, and Serbia in the Total Population Register. *

*Unexposed population (main comparison):* Individuals entering Sweden from January 1^st^ 1991 through December 31^st^ 2002 with a country of birth registered as*:* Moldavia, Romania, Slovakia, Slovenia, Check Republic, Czechoslovakia, and Hungary in the Total Population Register.*

*Unexposed population (secondary comparison):* Individuals entering Sweden from January 1^st^ 1991 through December 31^st^ 2002 with a country of birth registered as: Austria, Belgium, Denmark, Finland, France, Germany, Great Britain, Greece, Ireland, Iceland, Italy, Malta, The Netherlands, Norway, Portugal, Poland, Switzerland, and Spain.*

*Please note: Before final data bases are analyzed, and to confirm correct allocation to group the decision of about each country and their war status during this time period will be confirmed with a historian (who will not have access to the actual data).

*Unexposed population (tertiary comparison):* Individuals born and living in Sweden when matched to the exposed population on their entry date to Sweden (10 unexposed: 1 exposed), by age, sex, education and region.

**Ascertainment of outcome:**

Overall mortality, and cause-specific mortality as registered in the Causes of Death Register as: 1) Cardiovascular disease (ICD-10: I00-199), 2) Cancer (ICD-10: C00-C99) (later in sensitivity analysis divided into smoking and alcohol related cancer), 3) Suicide (ICD-10: X60-X84), 4) Injury-related deaths, not self-inflicted (ICD-10: V00-X58; X92-Y38), 4) All other causes. Similarly, we will also obtain all in-patient and outpatient diagnoses (from 2001) from the nationwide Patient- and Cancer Registers. We will focus on the diagnoses of: 1) Cardiovascular disease (ICD-10: I00-199), 2) Cancer (ICD-10: C00-C99), 3) Psychiatric disorders and suicide attempts (ICD-10: F00-F99; X60-X84; Y10-Y34), 4) various stress-related functional impairments, e.g. fibromyalgia (ICD-10: M79.7), low back pain (ICD-10: M54.5), tension headaches (ICD-10: G44), chronic fatigue (ICD-10: R53.8), non-cardiac chest pain (R07.8), irritable bowel syndrome (ICD-10: K58.9). We will further specifically disentangle stress-related disorders (e.g. PTSD; ICD-10: F43), anxiety (ICD-10: F40-F41) and depression (ICD-10: F32-F33) and corresponding psychotropic drug use in the Prescribed Drug Register (from 2005). Among immigrant children we will furthermore explore how refugee status influences educational attainment.

**Main co-variates:**

Main covariates include: age, sex, region of residency, period of immigration, marital status, education, and income as well as number of first-degree relatives. This information will be obtained from the Population Register, the Multi-Generation Register as well as the “Longitudinella integrationsdatabasen för sjukförsäkrings- och arbetsmarknadsstudier” (LISA) at Statistics Sweden. We added aggregate data on smoking and cancer- and cardiovascular disease-related death rates obtained from the World Health Organization's Global Health Observatory data repository; as well as data on mean suicide rates from Our World In Data.

*Follow-up:* We will follow all immigrants and native Swedish controls from the date of entry to Sweden, from 1991 through 2002, until date of occurrence of an index-health endpoint, death, emigration, or end of follow-up (Dec 31^st^ 2015), whichever occurs first.

**Data analysis:**

We will first contrast background characteristics of the refugee- and non-refugee immigrant populations to that of the native Swedes in terms of age at immigration, sex, calendar period of entry (1991-1995; 1996-1999; 2000-2002), attained education, occupation, annual income (obtained from LISA). Adjusting for relevant covariates, we will then use Cox proportional hazard regression models to assess the hazard ratios and 95% confidence intervals of major endpoints, contrasting incidence/mortality rates of the exposed vs. unexposed populations. We will further conduct stratified analysis, repeating our main analysis within different age groups, gender, period of immigration, and socio-economic groups. Lastly, we will test whether psychiatric disorders and other familial health hazards (family related factors) during the first years after refugee immigration mediate the associations to longer-term physical endpoints, e.g. cardiovascular disease and mortality.

**Significance:**

These studies may contribute to existing knowledge base on how refugee immigrants fare in a long-term perspective. Specifically, we hope to achieve:

- Better understanding of the links between war-refugee experience and health/functional outcomes: an under-studied area
- Better understanding of the role of psychiatric disorders as a possible risk factor for adverse health/functional outcomes in this population
- Conversely, identification of possible resilience factors (e.g. low levels of psychiatric disorders) that determine better health/functional outcomes
- Findings may indicate useful early targets post war-trauma (e.g. psychiatric symptoms) to minimize development of later adverse health/functional outcomes

**Collaborators**

We are a team of researchers primarily from clinical psychology and epidemiology at Karolinska Institutet.

Professor Unnur A. Valdimarsdóttir (Guest Professor, Institutionen för medicinsk epidemiologi och biostatistik, Karolinska Institutet and University of Iceland)

Professor Emily Holmes (Guest Professor, Inst. för klinisk neurovetenskap, Karolinska Institutet and MRC Cognition and Brain Sciences Unit, Cambridge, UK)

Professor Paul Lichtenstein (Head of Department, Institutionen för medicinsk epidemiologi och biostatistik, Karolinska Institutet)

Associate Professor Fang Fang (Institutionen för medicinsk epidemiologi och biostatistik, Karolinska Insitutet.

Dr. Li Yin (Biostatistician, Inst. Medicinsk epidemiologi och biostatistik, Karolinska Institutet.I

Professor Pär Sparén (Inst. Medicinsk epidemiologi och biostatistik, Karolinska Institutet.)

Professor Weimin Ye (Inst. Medicinsk epidemiologi och biostatistik, Karolinska Institutet)

Dr Filip Arnberg, Assistant Director, National Centre for Disaster Psychiatry, Uppsala University

**References**
