# Supplementary material for: Mortality and major disease risk among migrants of the 1991–2001 Balkan wars to Sweden: A register-based cohort study
Source: PLoS Med. 2020 Dec 1;17(12):e1003392. doi: 10.1371/journal.pmed.1003392 (PMC7707579; doi:10.1371/journal.pmed.1003392)
Supplement: S1 Table — (DOCX) [file pmed.1003392.s002.DOCX]

**S1 Table. Number of migrants with regard to whether they are migrants of the Balkan wars (exposed) or other European migrants (unexposed).**

| **Country** |  |
| --- | --- |
| **Exposed** | N |
| 1. Albania | 381 |
| 1. Bosnia-Herzegovina | 55,837 |
| 1. Croatia | 3,428 |
| 1. Macedonia | 1,790 |
| 1. Slovenia | 241 |
| 1. Yugoslavia | 43,093 |
| *Total* | *104,770* |
| **Unexposed** |  |
| 1. Austria | 1,148 |
| 1. Belgium | 1,127 |
| 1. Czech Republic | 552 |
| 1. Czechoslovakia | 811 |
| 1. Denmark | 18,083 |
| 1. Finland | 31,988 |
| 1. France | 5,233 |
| 1. Germany | 13,438 |
| 1. Great Britain | 11,520 |
| 1. Greece | 3,343 |
| 1. Hungary | 2,336 |
| 1. Iceland | 4,291 |
| 1. Ireland | 1,325 |
| 1. Italy | 2,882 |
| 1. Malta | 48 |
| 1. Moldova | 144 |
| 1. Netherlands | 3,502 |
| 1. Norway | 23,172 |
| 1. Poland | 11,779 |
| 1. Portugal | 725 |
| 1. Romania | 5,083 |
| 1. Slovak Republic | 362 |
| 1. Spain | 2,938 |
| 1. Switzerland | 1,600 |
| *Total* | *147,430* |
