# Supplementary material for: Mortality and major disease risk among migrants of the 1991–2001 Balkan wars to Sweden: A register-based cohort study
Source: PLoS Med. 2020 Dec 1;17(12):e1003392. doi: 10.1371/journal.pmed.1003392 (PMC7707579; doi:10.1371/journal.pmed.1003392)
Supplement: S2 Table — (DOCX) [file pmed.1003392.s003.DOCX]

**S2 Table. Prevalence of smoking in the year 2000 in Balkan war countries (exposed) and other European countries (unexposed) among individuals that were 15 years or older.***

| **Country** | **Males** | **Females** |  |  |  |
| --- | --- | --- | --- | --- | --- |
|  |  |  |  |  |  |
| **Exposed** |  |  |  |  |  |
| Bosnia-Herzegovina | 57.7 | 35.9 |  |  |  |
| Croatia | 40.3 | 28.0 |  |  |  |
| Macedonia** | 46.7 | 27.8 |  |  |  |
| Serbia | 54.6 | 41.2 |  |  |  |
| Slovenia | 29.9 | 22.6 |  |  |  |
| Albania | 56.9 | 11.6 |  |  |  |
| **Unexposed** |  |  |  |  |  |
| Czech Republic | 40.7 | 29.7 |  |  |  |
| Slovak Republic | 46.2 | 21.4 |  |  |  |
| Hungary | 46.1 | 34.4 |  |  |  |
| Moldova | 40.7 | 5.5 |  |  |  |
| Romania | 52.8 | 27.4 |  |  |  |
| Austria | 46.4 | 40.5 |  |  |  |
| Belgium | 35.6 | 24.3 |  |  |  |
| Denmark | 41.6 | 34.6 |  |  |  |
| Finland | 33.8 | 25.7 |  |  |  |
| France | 37.7 | 27.8 |  |  |  |
| Germany | 38.4 | 30.4 |  |  |  |
| United Kingdom | 31.3 | 28.9 |  |  |  |
| Greece | 63.0 | 42.4 |  |  |  |
| Iceland | 33.5 | 27.1 |  |  |  |
| Ireland | 33.1 | 30.5 |  |  |  |
| Italy | 32.9 | 20.2 |  |  |  |
| Malta | 38.8 | 26.9 |  |  |  |
| Netherlands | 36.7 | 30.7 |  |  |  |
| Norway | 43.1 | 41.0 |  |  |  |
| Portugal | 36.1 | 15.7 |  |  |  |
| Poland | 46.9 | 37.9 |  |  |  |
| Switzerland | 35.0 | 26.1 |  |  |  |
| Spain | 45.2 | 32.3 |  |  |  |
|  |  |  |  |  |  |
| *Smoking refers to daily or non-daily consumption of any form of tobacco, including cigarettes, cigars, and pipes. | | | |  |  |

** Data from 2009. Reference: http://www.euro.who.int/__data/assets/pdf_file/0011/312599/Tobacco-control-fact-sheet-TFYRofMacedonia.pdf
