# Supplementary material for: Mortality and major disease risk among migrants of the 1991–2001 Balkan wars to Sweden: A register-based cohort study
Source: PLoS Med. 2020 Dec 1;17(12):e1003392. doi: 10.1371/journal.pmed.1003392 (PMC7707579; doi:10.1371/journal.pmed.1003392)
Supplement: S3 Table — (DOCX) [file pmed.1003392.s004.DOCX]

**S3 Table. Cancer- and cardiovascular disease-related death rates in the year 1990 (age-standardized rates per 100,000 inhabitants) in Balkan war countries (exposed) vs. other European countries (unexposed).***

| **Country** | **Cancer** | **Cardiovascular disease** |
| --- | --- | --- |
| **Exposed** |  |  |
| 1. Albania | 122.04 | 400.27 |
| 1. Bosnia-Herzegovina | 163.53 | 572.16 |
| 1. Croatia | 197.66 | 525.45 |
| 1. Macedonia | 162.41 | 675.79 |
| 1. Serbia | 169.06 | 557.32 |
| 1. Slovenia | 186.30 | 438.11 |
| *Mean* | *166.83* | *528.18* |
| **Unexposed** |  |  |
| 1. Austria | 179.50 | 373.12 |
| 1. Belgium | 205.57 | 289.43 |
| 1. Czech Republic | 218.74 | 616.17 |
| 1. Denmark | 199.54 | 350.35 |
| 1. Finland | 156.66 | 395.98 |
| 1. France | 188.35 | 214.57 |
| 1. Germany | 179.00 | 383.65 |
| 1. Great Britain | 198.65 | 369.92 |
| 1. Greece | 161.41 | 348.87 |
| 1. Hungary | 237.14 | 595.24 |
| 1. Iceland | 160.79 | 284.11 |
| 1. Ireland | 192.42 | 392.04 |
| 1. Italy | 183.60 | 285.33 |
| 1. Malta | 165.65 | 405.67 |
| 1. Moldova | 149.85 | 709.28 |
| 1. Netherlands | 192.77 | 282.40 |
| 1. Norway | 163.35 | 320.13 |
| 1. Poland | 198.50 | 591.17 |
| 1. Portugal | 169.20 | 380.63 |
| 1. Romania | 132.08 | 625.98 |
| 1. Slovak Republic | 196.48 | 623.92 |
| 1. Spain | 162.66 | 258.49 |
| 1. Switzerland | 153.31 | 278.51 |
| *Mean* | *180.23* | *407.61* |

*Reference: <https://ourworldindata.org>
