# Supplementary material for: Mortality and major disease risk among migrants of the 1991–2001 Balkan wars to Sweden: A register-based cohort study
Source: PLoS Med. 2020 Dec 1;17(12):e1003392. doi: 10.1371/journal.pmed.1003392 (PMC7707579; doi:10.1371/journal.pmed.1003392)
Supplement: S4 Table — (DOCX) [file pmed.1003392.s005.DOCX]

**S4 Table. Mean suicide rate (age-adjusted rates of suicide per 100,000 inhabitants) in the year 1990 in the Balkan war countries (exposed) and other European countries (unexposed).***

| **Country** | **Suicide rate** |
| --- | --- |
| **Exposed** |  |
| 1. Albania | 4.24 |
| 1. Bosnia-Herzegovina | 12.28 |
| 1. Croatia | 23.06 |
| 1. Macedonia | 10.06 |
| 1. Slovenia | 30.18 |
| 1. Serbia | 19.27 |
| *Mean* | *16.52* |
| **Unexposed** |  |
| 1. Austria | 21.96 |
| 1. Belgium | 19.54 |
| 1. Czech Republic | 19.42 |
| 1. Denmark | 24.04 |
| 1. Finland | 28.05 |
| 1. France | 23.21 |
| 1. Germany | 15.13 |
| 1. Great Britain | 11.23 |
| 1. Greece | 3.88 |
| 1. Hungary | 35.65 |
| 1. Iceland | 15.28 |
| 1. Ireland | 10.71 |
| 1. Italy | 8.09 |
| 1. Malta | 5.65 |
| 1. Moldova | 19.47 |
| 1. Netherlands | 11.89 |
| 1. Norway | 15.07 |
| 1. Poland | 14.66 |
| 1. Portugal | 14.10 |
| 1. Romania | 9.88 |
| 1. Slovak Republic | 16.31 |
| 1. Spain | 7.94 |
| 1. Switzerland | 23.75 |
| *Mean* | *16.30* |

*Reference: https://ourworldindata.org/suicide
