# Supplementary material for: Mortality and major disease risk among migrants of the 1991–2001 Balkan wars to Sweden: A register-based cohort study
Source: PLoS Med. 2020 Dec 1;17(12):e1003392. doi: 10.1371/journal.pmed.1003392 (PMC7707579; doi:10.1371/journal.pmed.1003392)
Supplement: S5 Table — (DOCX) [file pmed.1003392.s006.DOCX]

**S5 Table. Smoking- and alcohol-related cancer incidence among migrants of the Balkan wars (exposed) vs. other European migrants (unexposed).**

|  | **Time since migration** | | | |  |
| --- | --- | --- | --- | --- | --- |
|  | **Year 0-1** | **Year 2-4** | **Year 5-9** | **Year 10+** | **Overall** |
| **Smoking-related cancer** |  |  |  |  |  |
| IR* (95% CI) for exposed | 78.9 (67.7-92.0) | 65.1 (56.6-74.8) | 84.0 (76.2-92.6) | 84.0 (76.4-92.2) | 79.5 (75.1-84.1) |
| IR (95% CI) for unexposed | 50.4 (42.5-59.7) | 40.2 (33.8-47.8) | 57.1 (50.5-64.6) | 56.5 (48.2-66.2) | 51.5 (47.7-55.5) |
| HR** (95 % CI) | 1.31 (0.96-1.79) | 1.36 (1.00-1.84) | 1.05 (0.85-1.30) | 1.53 (1.19-1.98) | 1.24 (1.09-1.42) |
| P value for HR | 0.095 | 0.051 | 0.648 | < 0.001 | 0.001 |
| **Alcohol-related cancer** |  |  |  |  |  |
| IR (95% CI) for exposed | 66.4 (56.2-78.5) | 50.0 (42.6-58.6) | 65.6 (58.8-73.2) | 83.8 (76.3-92.0) | 68.8 (64.8-73.1) |
| IR (95% CI) for unexposed | 48.7 (41.0-58.0) | 44.6 (37.9-52.6) | 58.9 (52.2-66.5) | 80.2 (70.2-91.7) | 57.8 (53.8-62.1) |
| HR** (95% CI) | 1.05 (0.79-1.41) | 1.06 (0.80-1-41) | 1.08 (0.87-1.32) | 1.16 (0.95-1.42) | 1.07 (0.95-1.20) |
| P value for HR | 0.728 | 0.685 | 0.489 | 0.146 | 0.257 |
| **Cancer not related to smoking and alcohol** |  |  |  |  |  |
| IR (95% CI) for exposed | 115.0 (101.3-130.6) | 92.4 (82.2-103.8) | 108.6 (99.7-118.3) | 115.8 (107.0-125.5) | 108.7 (103.6-114.1) |
| IR (95% CI) for unexposed | 86.6 (76.0-98.6) | 95.5 (85.4-106.9) | 93.2 (84.7-102.6) | 107.7 (96.0-120.9) | 95.5 (90.3-100.9) |
| HR** (95% CI) | 1.22 (0.95-1.57) | 1.05 (0.84-1.32) | 1.28 (1.06-1.53) | 1,29 (1.06-1.56) | 1.19 (1.07-1.32) |
| P value for HR | 0.120 | 0.661 | 0.009 | 0.011 | 0.001 |

*Incidence rates per 100,000 person-years; **Adjusting for sex, education, age at migration, smoking (country specific prevalence), and calendar period of immigration
