# Supplementary material for: Mortality and major disease risk among migrants of the 1991–2001 Balkan wars to Sweden: A register-based cohort study
Source: PLoS Med. 2020 Dec 1;17(12):e1003392. doi: 10.1371/journal.pmed.1003392 (PMC7707579; doi:10.1371/journal.pmed.1003392)
Supplement: S6 Table — (DOCX) [file pmed.1003392.s007.DOCX]

**S6 Table. Mortality and morbidity rates among migrants of the Balkan wars (exposed) compared to other European migrants (unexposed), with regard to whether individuals with missing education level are included.**

|  | Including *missing education* variable | Excluding *missing education* variable |
| --- | --- | --- |
|  | HR (95% CI*); p-value | HR (95% CI*); p-value |
| **Mortality** |  |  |
| Overall | 1.20 (1.14-1.27); p< 0.001 | 1.17 (1.10-1.24); p < 0.001 |
| Suicide | 0.68 (0.48-0.96); p = 0.030 | 0.62 (0.43-0.88); p = 0.008 |
| Cardiovascular disease^†^ | 1.45 (1.29-1.62); p< 0.001 | 1.43 (1.27-1.60); p < 0.001 |
| Cancers^††^ | 1.27 (1.15-1.41); p< 0.001 | 1.21 (1.09-1.35); p < 0.001 |
| **Morbidity** |  |  |
| Psychiatric diseases | 1.19 (1.14-1.23); p< 0.001 | 1.13 (1.09-1.18); p < 0.001 |
| Post-traumatic stress disorder | 9.33 (7.96-10.94); p< 0.001 | 7.43 (6.29-8.76); p < 0.001 |
| Suicide attempts | 0.57 (0.51-0.65); p< 0.001 | 0.54 (0.48-0.61); p < 0.001 |
| Cardiovascular disease^†^ | 1.39 (1.34-1.43); p< 0.001 | 1.37 (1.33-1.42); p < 0.001 |
| Cancer^††^ | 1.16 (1.08-1.24); p< 0.001 | 1.14 (1.07-1.23); p < 0.001 |

*Adjusting for sex, education, age at immigration, smoking (country specific prevalence) and calendar period of immigration.

^†^ Adjusted also for baseline CVD mortality.

^††^ Adjusted also for baseline cancer.
