# Supplementary material for: Mortality and major disease risk among migrants of the 1991–2001 Balkan wars to Sweden: A register-based cohort study
Source: PLoS Med. 2020 Dec 1;17(12):e1003392. doi: 10.1371/journal.pmed.1003392 (PMC7707579; doi:10.1371/journal.pmed.1003392)
Supplement: S1 Text STROBE checklist — (DOCX) [file pmed.1003392.s008.DOCX]

**S1. STROBE Statement—checklist of items that should be included in reports of observational studies.**

|  | Item No | Recommendation |  | Page number | |  | | |
| --- | --- | --- | --- | --- | --- | --- | --- | --- |
| **Title and abstract** | 1 | (*a*) Indicate the study’s design with a commonly used term in the title or the abstract |  | Title page | |  | | |
|  |  | (*b*) Provide in the abstract an informative and balanced summary of what was done and what was found |  | Abstract | |  | | |
| Introduction | | |  |  |  | |  |  |
| Background/rationale | 2 | Explain the scientific background and rationale for the investigation being reported |  | Introduction para 1-2 | |  | | |
| Objectives | 3 | State specific objectives, including any prespecified hypotheses |  | Introduction para 3 | |  | | |
| Methods | | |  |  |  | |  |  |
| Study design | 4 | Present key elements of study design early in the paper |  | Methods | |  | | |
| Setting | 5 | Describe the setting, locations, and relevant dates, including periods of recruitment, exposure, follow-up, and data collection |  | Methods: Study population and design | |  | | |
| Participants | 6 | (*a*) *Cohort study*—Give the eligibility criteria, and the sources and methods of selection of participants. Describe methods of follow-up |  | Methods: Study population and design | |  | | |
|  |  | (*b*) *Cohort study*—For matched studies, give matching criteria and number of exposed and unexposed |  | N/A | |  | | |
| Variables | 7 | Clearly define all outcomes, exposures, predictors, potential confounders, and effect modifiers. Give diagnostic criteria, if applicable |  | Methods: para 1-8 | |  | | |
| Data sources/ measurement | 8 | For each variable of interest, give sources of data and details of methods of assessment (measurement). Describe comparability of assessment methods if there is more than one group |  | Methods: para 3-8 | |  | | |
| Bias | 9 | Describe any efforts to address potential sources of bias |  | Statistical analysis | |  | | |
| Study size | 10 | Explain how the study size was arrived at |  | Methods: para 2 | |  | | |
| Quantitative variables | 11 | Explain how quantitative variables were handled in the analyses. If applicable, describe which groupings were chosen and why |  | Statistical analysis | |  | | |
| Statistical methods | 12 | (*a*) Describe all statistical methods, including those used to control for confounding |  | Statistical analysis: para 1-4 | |  | | |
|  |  | (*b*) Describe any methods used to examine subgroups and interactions |  | Statistical analysis: para 3-4 | |  | | |
|  |  | (*c*) Explain how missing data were addressed |  | Statistical analysis: para 1 | |  | | |
|  |  | (*d*) *Cohort study*—If applicable, explain how loss to follow-up was addressed |  | Methods: para 2 | |  | | |
|  |  | (*e*) Describe any sensitivity analyses |  | Methods: para 3-4 |  | | |  |

**Results**

| Participants | 13* | (a) Report numbers of individuals at each stage of study—eg numbers potentially eligible, examined for eligibility, confirmed eligible, included in the study, completing follow-up, and analysed | Results Table 1 |
| --- | --- | --- | --- |
|  |  | (b) Give reasons for non-participation at each stage | N/A |
|  |  | (c) Consider use of a flow diagram | N/A |
| Descriptive data | 14* | (a) Give characteristics of study participants (eg demographic, clinical, social) and information on exposures and potential confounders | Results para 1,  Table 1 |
|  |  | (b) Indicate number of participants with missing data for each variable of interest | Results Table 1 |
|  |  | (c) *Cohort study*—Summarise follow-up time (eg, average and total amount) | Results para 1-2; Table 1 |
| Outcome data | 15* | *Cohort study*—Report numbers of outcome events or summary measures over time | Results Table 2-5 |
|  |  | *Case-control study—*Report numbers in each exposure category, or summary measures of exposure | N/A |
|  |  | *Cross-sectional study—*Report numbers of outcome events or summary measures | N/A |
| Main results | 16 | (*a*) Give unadjusted estimates and, if applicable, confounder-adjusted estimates and their precision (eg, 95% confidence interval). Make clear which confounders were adjusted for and why they were included | Results para 4-15, Table 2-5 |
|  |  | (*b*) Report category boundaries when continuous variables were categorized | Results, Table 2 & 4 |
|  |  | (*c*) If relevant, consider translating estimates of relative risk into absolute risk for a meaningful time period | N/A |
| Other analyses | 17 | Report other analyses done—eg analyses of subgroups and interactions, and sensitivity analyses | Results para 4,9-11,13-14  Table 2, 4, S5, S6 |
| **Key results** | 18 | Summarise key results with reference to study objectives | Discussion para 1 |
| Limitations | 19 | Discuss limitations of the study, taking into account sources of potential bias or imprecision. Discuss both direction and magnitude of any potential bias | Discussion para 2 |
| Interpretation | 20 | Give a cautious overall interpretation of results considering objectives, limitations, multiplicity of analyses, results from similar studies, and other relevant evidence | Discussion para 4-9 |
| Generalisability | 21 | Discuss the generalisability (external validity) of the study results | Discussion para 2 |
| Funding | 22 | Give the source of funding and the role of the funders for the present study and, if applicable, for the original study on which the present article is based | Funding |
