# Supplementary material for: Mortality and major disease risk among migrants of the 1991–2001 Balkan wars to Sweden: A register-based cohort study
Source: PLoS Med. 2020 Dec 1;17(12):e1003392. doi: 10.1371/journal.pmed.1003392 (PMC7707579; doi:10.1371/journal.pmed.1003392)
Supplement: Attachment — Submitted filename: Comments to author.docx [file pmed.1003392.s009.docx]

**Comments to author**

This is an important and well-performed register-based investigation on the risk of mortality and major disease risk among migrants of the 1991-2001 Balkan wars to Sweden compare to migrants from other European counties to Sweden during the same period. The strengths of the study includes the use of comprehensive registers and careful statistical analyses. The manuscript is well written and easy to read.

I have only minor comments:

METHODS

1. They authors should describe any work/effort validating the information source. How accurate is the information in this database, how complete are the data; are they equally complete throughout the study period?
2. Line 85, ICD-10 for CVD diagnosis is I00-I99, not 100-199
3. Line 94 and Line 97, ICD-7 code of 150 is “esophagus”
4. Line 95, ICD-7 code for lung cancer 162-163, code 161 is a diagnosis of larynx, the author has defined it as a separate subtype (Line 98).
5. Line 97, ICD-7 code for liver cancer are 155 and 156
6. Line 107-108, please indicate first degree relatives in the methods; as the authors have mentioned in Table 4, siblings, parents, and children.
7. Please clarify how the rate of morbidities and mortalities was calculated, it was for per 100 000 inhabitants or per 100 000 person years.

RESULTS

8. Please add a footnote to indicate incidence rates (IR) in Table 1, 2 and Supplementary Table 5, for per 100 000 inhabitants, or per 100 000 person years.

DISCUSSIONS AND CONCLUSION

9. It should be noted that cancer survival in Sweden overall is internationally favorable. The findings that some groups with low cancer incidence may have even a more favorable survival. Even the study has controlled by the variable of residence time and country-level cancer and related rates, the possibility of immigrants diagnosed with a cancer in the earlier stage may move back to their home country, while severely ill patients stay in host country, causing bias in Hazard rates.
10. Suggest how your findings are useful for health care workers (or policy makers)? What might they do with this information?
