# Supplementary material for: Mortality and major disease risk among migrants of the 1991–2001 Balkan wars to Sweden: A register-based cohort study
Source: PLoS Med. 2020 Dec 1;17(12):e1003392. doi: 10.1371/journal.pmed.1003392 (PMC7707579; doi:10.1371/journal.pmed.1003392)
Supplement: Attachment — Submitted filename: Response_to_Reviewers.DOCX [file pmed.1003392.s010.DOCX]

**Response to Reviewers**

**Comment #1:** Please revise your title according to PLOS Medicine's style. Your title must be nondeclarative and not a question. It should begin with main concept if possible. Please place the study design ("A randomized controlled trial," "A retrospective study," "A modelling study," etc.) in the subtitle (ie, after a colon). eg, "Mortality and major disease risk among migrants of the 1991-2001 Balkan wars to Sweden: register-based cohort study".

**Response:** Thank you, the title of the manuscript has been changed to "Mortality and major disease risk among migrants of the 1991-2001 Balkan wars to Sweden: A register-based cohort study".

**Comment #2:**  At this stage, we ask that you include a short, non-technical Author Summary of your research to make findings accessible to a wide audience that includes both scientists and non-scientists. The Author Summary should immediately follow the Abstract in your revised manuscript. This text is subject to editorial change and should be distinct from the scientific abstract. Please see our author guidelines for more information: <https://eur02.safelinks.protection.outlook.com/?url=https%3A%2F%2Fjournals.plos.org%2Fplosmedicine%2Fs%2Frevising-your-manuscript%23loc-author-summary&amp;data=01%7C01%7Ceddat%40hi.is%7Cdad066674eb94391647708d7e5101a13%7C09fa5f0e211846568529677ed8fdbe78%7C0&amp;sdata=r26Cc4%2FxZNylnMQoy53YocaP%2Bn%2Bwmyoot2feL9iQazU%3D&amp;reserved=0>

**Response:** Thank you, this has been added to the manuscript (pages 2-3, lines 81-112).

**Comment #3:** Please ensure that the study is reported according to the STROBE guideline, and include the completed STROBE checklist as Supporting Information. When completing the checklist, please use section and paragraph numbers, rather than page numbers. Please add the following statement, or similar, to the Methods: "This study is reported as per the Strengthening the Reporting of Observational Studies in Epidemiology (STROBE) guideline (S1 Checklist)."

**Response:** The Strobe checklist has been added as Supporting Information and the statement has been added to the methods chapter (page 6, line 165-167). In accordance to the Strobe guidelines, we have added information about the total amount of follow-up time to the results section (page 10, lines 283-284). We have also added to the methods section information about the quality of the Swedish Causes of Death Register (page 6, lines 174-175), the Swedish National Patient Register (page 6, line 181) and the PTSD diagnoses in Swedish register data (page 6, lines 183-185). Information about the external validity of the study has also been added to the discussion chapter (page 22, lines 427-429).

**Comment #4:** As the study is observational, it isn't straight forward to infer causality; in most of the manuscript text, this is appropriately presented but there are a few places where the authors mention "elevated/increased risk" or "reduced risk" which does seem to imply the association is causal and the language could be fine-tuned in such instances (ie "associated with increased risk").

**Response:** Thank you, we totally agree. We have now edited sentences that previously could be interpreted as inferring causality in the abstract (page 1, lines 15, 18-19), results section (page 11, lines 292-293; 299-300; 302-303; page 15, lines 330; page 16 line 338, 345, 354; 358; page 17, line 365; 368; 373) and discussion section (page 21, line 391-392, page 23, line 468; page 24, lines 495-497; page 25, 513-514).

**Reviewer #1**

**Comment #1:** One big issue is the use of linear regression. Since there are a lot of people from each of a bunch of countries, the assumption of independent errors that linear regression makes will be violated. One method of dealing with this is a multilevel model. In addition, this allows clearer use of both individual and country-level variables.

**Response:** We want to point out that our main statistical approach throughout the manuscript is using Cox proportional hazard regression models to calculate hazard ratios, comparing outcome event rate among migrants of the Balkan wars to that among other European migrants. Country-level information on smoking prevalence, and CVD and cancer mortality rates (in our mortality estimation) was only used in the full multivariable models and these did not significantly affect our results. Linear regression was only used to test if HRs for CVD and psychiatric morbidity were higher among those with a late period of immigration, compared with those with an earlier period of immigration, and those with no 1^st^ degree relatives in Sweden, compared with those with 1^st^ degree relatives in Sweden. Here, we could also have used multilevel models, where a random effect would be specified for the variability of so many countries. On the other hand, it was difficult to incorporate the country-level variables such as the smoking status into the random effect so we decided to use our original analytic strategy.

**Comment #2:** In addition, Line 106-7 Do not categorize independent variables. In *Regression Modeling Strategies* Frank Harrell lists 11 problems with doing this and sums up "Nothing could be more disastrous". I wrote a blog post illustrating some of the problems : <https://eur02.safelinks.protection.outlook.com/?url=https%3A%2F%2Fmedium.com%2F%40peterflom%2Fwhat-happens-when-we-categorize-an-independent-variable-in-regression-77d4c5862b6c&amp;data=01%7C01%7Ceddat%40hi.is%7Cdad066674eb94391647708d7e5101a13%7C09fa5f0e211846568529677ed8fdbe78%7C0&amp;sdata=ZDDnGi6mYVzJrPH0laAZ9i5i7D5zlGB5ctRldWV6krI%3D&amp;reserved=0>

**Response:** Thank you, we agree with the reviewer on this point. In the analysis, we had adjusted for sex, education, age at immigration, smoking and calendar period of immigration. Age at immigration was continuous. As the reviewer suggests, we have now adjusted for calendar period of immigration as a continuous variable in Tables 2-5 and Supplementary Tables S5 and S6. We have stated that calendar period of immigration is modelled as a continuous variable in all models in the statistical analysis section (page 9, line 260). All results in the manuscript have been updated accordingly.

**Reviewer #2**

The paper addresses a relevant health problem and the data used together with the analytic strategy adds to the literature. The topic as well as the data-set itself is very complex and this is a good attempt to attack the issues at hand. To this reviewer's mind, there are some points that could be sharpened in the paper.

**Response:** Thank you for the encouraging comments.

**Comment #1:** The background and the conclusion in the abstract should - as the methods part does - make clear that this is a comparison between different groups of immigrants.

**Response:** Thank you, this has been added to the background (page 1, line 5-6) and conclusion (page 2, line 77) in the abstract.

**Comment #2:** The authors present complex models first and then later return to stratified analyses (but also there we see only adjusted models). I would much have preferred to from the beginning see more presentation of the crude data, e.g. starting with something like table 4 displaying incidence rates, HR:s in unadjusted and then adjusted models in strata as in table 4. Then proceed to the multivariate models. Likewise, it would be easier to understand the data and follow the line of interpretation if we in the modeling could see what happens in different steps of the adjustment. This need not be covariate by covariate, but could be in "chunks" of co-variates. The present results-section does not convince the reader that the models presented are the adequate ones and does not permit assessment of effect-modification and alike phenomena.

**Response:** We agree with the reviewer that it is a good idea to present the modeling in steps. Accordingly, we now first present crude data and then proceed to the multivariate models in Tables 2 and 4.

**Comment #3:** Persons with missing data on education is an extreme group judging from table 4. It is not clear in the methods part how the missing category was handled and if the choice of method here influences the results in a major way. Have the authors considered an imputation here or some more advanced strategy to find out what is happening?

**Response:** As 4% of individuals in our study had missing information on education attainment, we opted to present this group separately as people with missing education may be different from others. Specifically, the degree of missing on education status is higher than in other available covariates, e.g. gender, age on entry, gender, entry period, first-degree relatives at entry, and country-level smoking. Missing is not random on educational level given these covariates and will thus not sufficiently be addressed with tools such as multiple imputation. Therefore, we treated this group as a separate group at the cost of some efficiency. To test the influence of our approach, we conducted a sensitivity analysis where we compared HR among those with known educational level (omitting the group with missing education) to the HRs where we treat missing education as separate group. Our results indicate that the missing education has little influence on our results. The results of our sensitivity analysis are now presented in the Supplementary Table S6 and discussed in the results section (page 17, lines 375-376).

**Comment #4:** The tables are very unclear about what a "rate" is. E.g. table 4 shows hazard ratios, not rates, the other tables show both incidence rates and hazard ratios. It is very difficult to find out the enumeration of the denominators for the rates.

**Response:** We have added information about incidence rates to Table 4 and added a new table showing mortality rates in Balkan war migrants compared with other European migrants, across strata of sociodemographic and lifestyle-related characteristics (Table 2).

**Comment #5:** It is very good that we for many tables see both incidence rates and the hazard ratio. This in turn could lead to a part in the discussion to show what the absolute health impact is likely to be in say 1000 individuals during five years of observation.

**Response:** We agree with the reviewer on the importance of presenting incidence rates and have now done so throughout Tables 2 and 4 (see revised manuscript pages 12-13 and 18-19).

**Comment #6:** The discussion could be better structured. The study, say the authors, "offers a unique opportunity to examine the health consequences of additional stressors brought on by war exposure". The critical question is then if the design, data, analysis and results reflect a causal pathway for this, or if the findings are a result of other things such as data handling, selection of who migrates, or exposures in the home-country. Some of the hazard ratios are quite low and could be the result of bias rather than causal. The discussion enters this discussion but only partly and fragmented. Mostly, the authors use a rather strong "causal language" - which admittedly could be used around psychiatric disorders but is more questionable for e.g. cancer. A clearer structure could probably also lead to a shortening, which would put the message across clearer.

Response: Thank you for your suggestion. We have now altered language inferring causality in the abstract (page 1, lines 15, 18-19), results section (page 11, lines 292-293; 299-300; 302-303; page 15, lines 330; page 16 line 338, 345, 354; 358; page 17, line 365; 368; 373) and discussion section (page 21, line 391-392, page 23, line 468; page 24, lines 495-497; page 25, 513-514).

We have also restructured our discussion, moving the section about the limitations of the study after our summary of the main findings (page 21-22, lines 397-429) and added more information about potential biases in the study (page 21-22 lines 398-421).

**Comment #8:** In the conclusion, line 315-321 is a repetition of the main findings and unnecessary. What comes after are consequences of the findings and the interpretation, which is a good final note. Could more of this be "transplanted" to the abstract?

**Response:** This repetition of the main findings has been removed and information on the consequences of the findings has been added to the abstract as suggested (page 2, line 79-80).

**Review #3**

This is an important and well-performed register-based investigation on the risk of mortality and major disease risk among migrants of the 1991-2001 Balkan wars to Sweden compare to migrants from other European counties to Sweden during the same period. The strengths of the study includes the use of comprehensive registers and careful statistical analyses. The manuscript is well written and easy to read.

**Response:** Thank you for the encouraging comments.

**Comment #1:** They authors should describe any work/effort validating the information source. How accurate is the information in this database, how complete are the data; are they equally complete throughout the study period?

**Response:** Thank you for this suggestion, we have added to the methods section information about the validity/quality of the the Swedish Causes of Death Register (page 6, line 174-175), the Swedish National Patient Register (page 6, line 181) including the validity of the PTSD diagnoses in this register using data from the Region of Stockholm (page 6, lines 183-185).

**Comment #2:** Line 85, ICD-10 for CVD diagnosis is I00-I99, not 100-199

**Response:** This has been corrected (page 6, line 173).

**Comment #3:** Line 94 and Line 97, ICD-7 code of 150 is “esophagus”

**Response:** This has been corrected (page 7, line 195).

**Comment #4:** Line 95, ICD-7 code for lung cancer 162-163, code 161 is a diagnosis of larynx, the author has defined it as a separate subtype (Line 98).

**Response:** This has been corrected (page 7, line 196).

**Comment #5:** Line 97, ICD-7 code for liver cancer are 155 and 156

**Response:** Sub-analysis were only conducted for primary liver cancer. This has been clarified in the manuscript (page 7, line 198).

**Comment #6:** Line 107-108, please indicate first degree relatives in the methods; as the authors have mentioned in Table 4, siblings, parents, and children.

**Response:** We have added information about first degree relatives in the methods section (page 7, lines 206-207).

**Comment #7:** Please clarify how the rate of morbidities and mortalities was calculated, it was for per 100 000 inhabitants or per 100 000 person years.

**Response:** The rate of morbidities and mortalities was calculated per 100 000 person years. This has been added to the statistical analysis section (page 8, line 238).

**Comment #8:** Please add a footnote to indicate incidence rates (IR) in Table 1, 2 and Supplementary Table 5, for per 100 000 inhabitants, or per 100 000 person years.

**Response:** Thank you, we have added footnotes in Tables 2-5 and Supplementary Table S5 indicating that incidence rates are per 100,000 person-years. Please not that incidence rates are not presented in Table 1.

**Comment #9:** It should be noted that cancer survival in Sweden overall is internationally favorable. The findings that some groups with low cancer incidence may have even a more favorable survival. Even the study has controlled by the variable of residence time and country-level cancer and related rates, the possibility of immigrants diagnosed with a cancer in the earlier stage may move back to their home country, while severely ill patients stay in host country, causing bias in Hazard rates.

**Response:** To be clear, our main comparison is cancer incidence and mortality across two immigrant groups, one exposed and the other unexposed to the Balkan wars. In our estimation of cancer mortality, we have not taken into account stage of diagnosis and it is possible that migrants of the Balkan wars were diagnosed at a later stage than other migrants. However, our results on cancer incidence do not suggest lower detection of cancers among Balkan war migrants. Yet, we have added information about this potential bias to the discussion chapter (page 21, lines 400-402).

**Comment #10:** Suggest how your findings are useful for health care workers (or policy makers)? What might they do with this information?

**Response:** Thank you, this has been added to the discussion section (page 26, lines 568-571).
