# Supplementary material for: Mortality and major disease risk among migrants of the 1991–2001 Balkan wars to Sweden: A register-based cohort study
Source: PLoS Med. 2020 Dec 1;17(12):e1003392. doi: 10.1371/journal.pmed.1003392 (PMC7707579; doi:10.1371/journal.pmed.1003392)
Supplement: Attachment — Submitted filename: Comments to authorXL2.docx [file pmed.1003392.s011.docx]

**Comments:**

The authors have satisfactorily responded to all my questions and made the necessary changes to the manuscript. I have no additional comments.
